# Virus-induced exacerbations in asthma and COPD

**DOI:** 10.3389/fmicb.2013.00293

**Published:** 2013-10-01

**Authors:** Daisuke Kurai, Takeshi Saraya, Haruyuki Ishii, Hajime Takizawa

**Affiliations:** Department of Respiratory Medicine, Kyorin University School of MedicineMitaka, Tokyo, Japan

**Keywords:** asthma, COPD, respiratory virus, exacerbation, overlap syndrome, human rhinovirus, respiratory syncytial virus

## Abstract

Chronic obstructive pulmonary disease (COPD) is characterized by chronic airway inflammation and/or airflow limitation due to pulmonary emphysema. Chronic bronchitis, pulmonary emphysema, and bronchial asthma may all be associated with airflow limitation; therefore, exacerbation of asthma may be associated with the pathophysiology of COPD. Furthermore, recent studies have suggested that the exacerbation of asthma, namely virus-induced asthma, may be associated with a wide variety of respiratory viruses. COPD and asthma have different underlying pathophysiological processes and thus require individual therapies. Exacerbation of both COPD and asthma, which are basically defined and diagnosed by clinical symptoms, is associated with a rapid decline in lung function and increased mortality. Similar pathogens, including human rhinovirus, respiratory syncytial virus, influenza virus, parainfluenza virus, and coronavirus, are also frequently detected during exacerbation of asthma and/or COPD. Immune response to respiratory viral infections, which may be related to the severity of exacerbation in each disease, varies in patients with both COPD and asthma. In this regard, it is crucial to recognize and understand both the similarities and differences of clinical features in patients with COPD and/or asthma associated with respiratory viral infections, especially in the exacerbative stage. In relation to definition, epidemiology, and pathophysiology, this review aims to summarize current knowledge concerning exacerbation of both COPD and asthma by focusing on the clinical significance of associated respiratory virus infections.

## INTRODUCTION

Asthma and chronic obstructive pulmonary disease (COPD) are very common inflammatory diseases of the airways. The World Health Organization (WHO) estimates that asthma accounts for 1 in every 250 deaths worldwide (O’Sullivan, 2005). The prevalence of asthma in developed countries is approximately 10% in adults and even higher in children, while in developing countries, the prevalence is lower but increasing rapidly (Barnes, 2008). In the case of COPD, WHO consensus reports forecast that this disorder will be ranked the third cause of mortality in the world by 2020 (Global initiative for chronic obstructive lung disease [GOLD], 2013^1^). Acute deterioration of symptoms and lung function, which often results in respiratory failure, is a so-called “exacerbation,” and it is an important and severe social and medical burden in both diseases.

**FIGURE 1:**
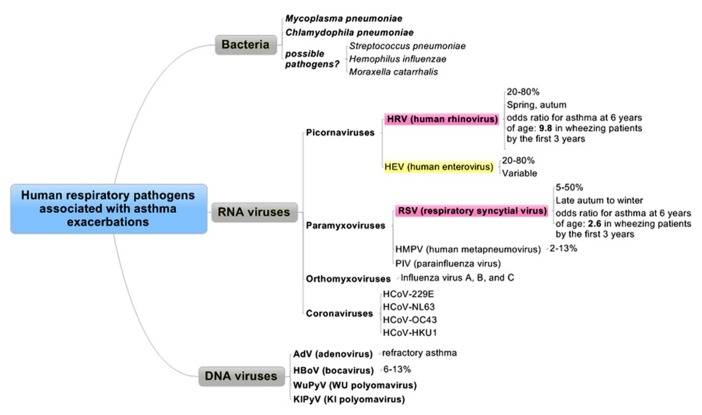
**Human respiratory pathogens associated with asthma exacerbations.** Pathogens are divided into three categories as bacteria, RNA viruses, or DNA viruses. Red and yellow columns are the most relevant pathogens in order.

**FIGURE 2:**
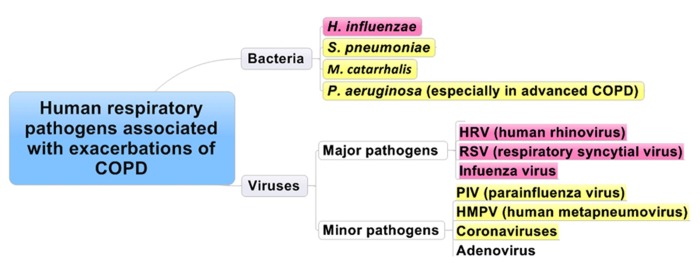
**Human respiratory pathogens associated with exacerbations of COPD.** Red and yellow columns are the most relevant pathogens in order.

Respiratory viral infections are common and usually self-limiting illnesses in healthy adults and a major cause of exacerbations in patients with asthma (**Figure 1**) and/or COPD (**Figure 2**).

This review aims to summarize the clinical aspects of exacerbations in asthma and COPD from the perspective of the definition of exacerbations, epidemiology, and pathophysiology, with a special focus on the clinical significance of the presence of respiratory viruses.

## PATHOPHYSIOLOGY OF ASTHMA AND COPD

Asthma and COPD are prevalent chronic pulmonary diseases characterized by chronic airway inflammation and airflow limitation. The differences between the two diseases are mainly the cellular and molecular features of airway inflammation and the degree of reversibility of airway flow limitation. Generally, reversibility of airflow limitation is incomplete in COPD, while that in asthma can be complete. Airway inflammation in asthma is characterized by allergic phenotypes, such as dense infiltration of eosinophils and T helper type 2 lymphocytes, associated with atopic status, while that of COPD is mainly accumulation of neutrophils, CD8-positive cytotoxic T cells, and activated macrophages, which are caused by inhalation of harmful substances, such as smoking. With respect to the site of inflammation, asthma involves predominantly larger airways, while in COPD, inflammation affects predominantly small airways and the lung parenchyma, characterized as irreversible airway narrowing because of fibrosis around the small airways or destruction of alveolar walls with protease-mediated degradation (Barnes, 2008). Of note, neutrophilic infiltration could be recognized in bronchial biopsied specimens as well as eosinophils in severe refractory asthma (Wenzel et al., 1999).

## DIFFERENCES AND SIMILARITIES BETWEEN ASTHMA AND COPD

As described above, asthma is typically characterized by chronic allergic inflammatory airway inflammation associated with airway hyperresponsiveness that leads to recurrent episodes of bronchial obstruction. In contrast, COPD is characterized by persistent airflow limitation that is usually progressive and ultimately results in respiratory failure. Therefore, it is not difficult to differentiate clinically between the two disorders. However, determining whether a patient has asthma or an exacerbation of COPD is often difficult, because of their clinical similarity. The **Table 1** summarizes the differences between these two diseases, and Tokuda and Miyagi (2007) provided an excellent review of rapid physical diagnosis for COPD patients that focused on inspection, palpation, percussion, auscultation, special maneuvers, and vital signs.

**Table 1:** Differences between asthma and COPD.

	Asthma	COPD
Age at onset	At any age (usually <40 years)	Usually >40 years
Smoking history	Possible	Usually >10 pack-years
Cough at exacerbation	Usually between 2 and 6 am	Gradual increase
Sputum production	Infrequent	Common
Allergy (eczema or allergic rhinitis)	Common	Infrequent
**Airway inflammation**		
Main portion	Large airways	Small airways
Pathophysiology	Basement-membrane thickening	Fibrosis of small airways
	Increased airway smooth muscle	Destruction of alveolar walls
Bronchial biopsies	Th2-dominant T cells	Th1-dominant T cells and type1 CTL
	Eosinophils, activated mast cells	Neutrophils and Mφ
Reversibility (peak flow results)	Normalize with time	May improve, but do not normalize
Family history	Common	Uncommon

*CTL, cytotoxic T cell; Mφ , macrophage.
*

On physical examination, the sound of an expiratory wheeze is identical in asthma, COPD, congestive heart failure, and pneumonia, and it cannot be used to distinguish among these conditions (Kaplan et al., 2009). Thus, physical examination is relatively insensitive for the diagnosis of asthma, but COPD has its characteristic physical findings (Tokuda and Miyagi, 2007) that could be useful in rapid differentiation from those of asthma.

Recent understanding of the innate immune system suggests that it may function independently of the adaptive immune system in some cases or synergistically in others, and the relative contributions of the two systems may explain the disease heterogeneity among asthmatic patients, which might occur in patients with COPD (Holtzman, 2012). It has long been argued that asthma, chronic bronchitis, and emphysema could be considered as different expressions of one disease entity. This view is called the “Dutch hypothesis” (Kraft, 2006), and it is still under debate, with no consensus about it. There are many similarities in asthma and COPD (Bleecker, 2004), and previous studies suggested that asthma may be a risk factor for the development of COPD (Silva et al., 2004), while the coexistence of asthma and COPD, so-called “overlap syndrome,” has recently been attracting attention. Overlap syndrome accounts for about 15–25% of obstructive airway disease (Louie et al., 2013) and shows more frequent or severe exacerbations and higher mortality than COPD alone (Hospers et al., 2000; Hardin et al., 2011). Furthermore, previous reports noted that exacerbations of asthma or COPD are associated with accelerated loss of lung function and quality of life and increased healthcare costs and mortality.

Thus, it is crucial to recognize and understand the clinical features of asthma and COPD patients, not only in the stable phase, but also in exacerbated phases associated with respiratory viral infections. Johnston and Sears (2006) reported that exacerbations of asthma and COPD appear to have a seasonal predilection in a similar fashion.

## VIRUS-INDUCED EXACERBATIONS IN ASTHMA AND COPD

### VIRUS-INDUCED EXACERBATIONS IN ASTHMA

**FIGURE 3:**
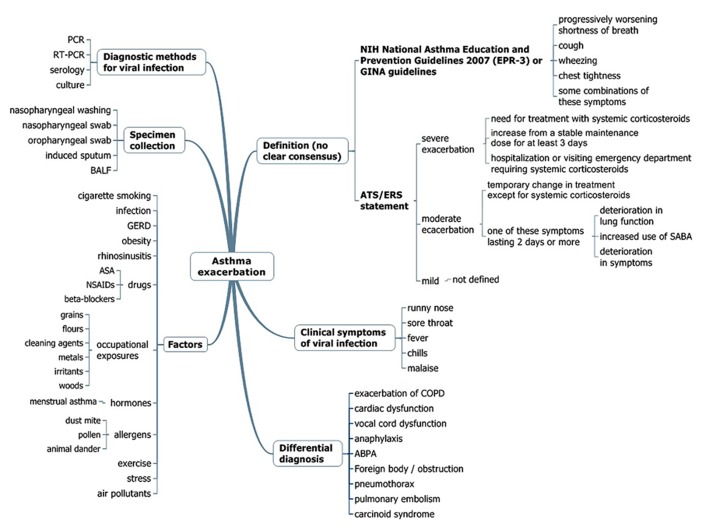
**Multidisciplinary assessment for asthma exacerbation.** PCR, polymerase-chain reaction; RT-PCR, real-time PCR; BALF, bronchoalveolar lavage fluid; GERD, gastroesophageal reflux disease; ASA, acetylsalicylic acid; NSAIDS, non-steroidal anti-inflammatory drugs; SABA, short-acting β-agonist inhalers; GINA, Global Initiative for Asthma; ABPA, allergic bronchopulmonary aspergillosis; EPR, expert panel report.

In bronchial asthma, acute exacerbation involves several issues (**Figure 3**), such as the definition of acute exacerbation of asthma, recognition of the clinical symptoms of respiratory tract infection (RTI), assessment of the risk factors for acute exacerbation, considering the possibility of other diseases (differential diagnosis), diagnostic methods, appropriate sample collection, and treatment or prevention. An older study showed that asthmatic patients had a 6.2 times greater chance of having viral RTIs than a control group (Abramson et al., 1994), while Corne et al. (2002) found that the detection rates of human rhinovirus (HRV) in asthmatic (10.1%) and healthy participants (8.5%) were similar. The term virus-induced exacerbation of asthma is not uncommon, but only a small number of such studies were prospective (Nicholson et al., 1993; Johnston et al., 1995). Furthermore, RTIs do not always lead to an exacerbation, and there is little evidence that treating or preventing the infection may cure or prevent an exacerbation. In this regard, we mainly discuss the details of “viral infection and exacerbation of asthma,” focusing on the accumulation of useful expertise for understanding this unfavorable condition in adult asthmatic patients.

#### Definitions of acute exacerbation of asthma

The diagnosis of asthma is usually defined based on history and variability of the peak expiratory flow rate (PEFR) and/or of forced expiratory volume in 1 s (FEV_1.0_) of at least 20%, either with therapy or spontaneously. There is no clear consensus definition for asthma exacerbation; clinical trials usually define a severe exacerbation as the need for treatment with systemic corticosteroids, hospital admission, or emergency treatment for worsening asthma, or a decrease in morning peak flow >25% baseline on two consecutive days (O’Byrne et al., 2001). According to the latest NIH National Asthma Education and Prevention Guidelines, asthma exacerbations are acute or subacute episodes of progressively worsening shortness of breath, cough, wheezing, and chest tightness, or some combination of these symptoms, characterized by decreases in expiratory airflow and objective measures of lung function (spirometry and peak flow) (National Asthma Education and Prevention Program, 2007), identical to the definition of the Global Initiative for Asthma guidelines (2012)^2^ (**Figure 3**). However, a joint task force of the American Thoracic Society and European Respiratory Society has recently defined asthma exacerbations as events characterized by a change from the patient’s previous status (Reddel et al., 2009). Severe exacerbations were defined as events that require urgent action to prevent hospitalization or death, whereas moderate exacerbations were defined as the status of an asthmatic patient who required a prompt change in treatment due to being outside the patient’s usual range of day-to-day asthma variation. Mild exacerbations are not defined because such events can be indistinguishable from loss of asthma control (Reddel et al., 2009; **Figure 3**).

#### Epidemiology

Asthma exacerbations are more common in female than in male patients, and the higher prevalence of asthma in adult women contrasts with the higher prevalence of asthma in male children (Bjornson and Mitchell, 2000). Between 14 and 45% of acute asthma exacerbations in children is thought to be related to viral RTIs. Although the incidence in adults is less clear, previous reports showed that RTIs associated with asthma exacerbation in adults ranged from 10–21% (Teichtahl et al., 1997) to 45–80% (Johnston et al., 1995; Atmar et al., 1998), of which 60% have HRV (Johnston et al., 1995; Atmar et al., 1998; Tan, 2005; **Figure 1**). Despite their widely differing designs, these studies suggest that viral infections are involved in about 50% of asthma exacerbations among adults and in probably substantially more childhood asthma exacerbations. Another report also showed that the virus most commonly implicated in asthma exacerbations appears to be HRV (Murray et al., 2004). In addition to HRV, other respiratory tract viruses, such as respiratory syncytial virus (RSV), influenza viruses, coronaviruses, human metapneumoviruses (HMPVs), parainfluenza viruses (PIVs), adenoviruses (AdVs), and bocaviruses, have all been detected in subjects with asthma exacerbations (Jackson and Johnston, 2010). In adults requiring hospital admission for an acute severe asthma exacerbation in a 1-year period, virus was identified in 29% of the subjects, with HRV and influenza A the most commonly identified infectious agents (Teichtahl et al., 1997).

#### Diagnosis of viral infection: diagnostic methods and sample collection

Molecular methods of viral detection have superior sensitivity and specificity compared to cell culture-based methods (McErlean et al., 2010). In the setting of acute exacerbation, the reverse-transcriptase polymerase chain reaction (RT-PCR) method can detect viruses in approximately 80% of wheezing episodes in school-aged children and in approximately one-half to three-quarters of acute wheezing episodes in adults (Jackson and Johnston, 2010). With respect to sample collections for viral detection, Xiang et al. (2002) reported that nasopharyngeal secretions and induced sputum during acute exacerbations of asthma in adult patients were equal, while Falsey et al. (2012) found that the diagnostic yields using RT-PCR for detection of any virus in adults hospitalized with respiratory illness were superior in sputum samples (36%) than in nose and throat swabs (23%). However, the study had some limitations in that no test for HRV or AdVs was used. Another report showed that the sensitivity rates for oropharyngeal swabs (OPS), nasopharyngeal swabs (NPS), and nasopharyngeal washings (NPW) obtained from hospitalized patients with acute febrile lower respiratory tract (LRT) infections were 54.2, 73.3, and 84.9%, respectively (for OPS vs. NPS and NPW, *p* < 0.00001; for NPS vs. NPW, *p* < 0.003; Lieberman et al., 2009). Taken together, these studies appear to suggest that induced sputum and NPS/NPW are better methods for identifying respiratory viruses. Regarding HRV, bronchoalveolar lavage (BAL) cells were positive for HRV RNA during infection in 80% of samples, whereas nasal lavage fluid was positive in the same patients in 100%, and BAL fluid was positive in only 37%. This suggests that HRV is able to infect the lower airways, and that HRV RNA is largely cell-associated (Murray et al., 2004).

#### Causes of acute asthma exacerbations

Eczema and a family history of asthma are the dominant non-infectious risk factors for pediatric asthma, while the triggers of adult-onset disease are less well defined. The causes for asthma exacerbation have been described and categorized. Of note, clinicians should recognize the seasonal trends for exacerbations of wheezing or asthma in adults, which occur 1–2 weeks later than in children, suggesting household transmission of the same strain (Johnston et al., 1996). HRV can be documented throughout the year, with a predilection for late spring and fall (Nagel et al., 2008), whereas RSV can be detected in late autumn to winter (**Figure 1**).

***Viruses***. The most important viruses relevant to asthma development are RSV and HRV. Jackson et al. (2008) demonstrated that, in a large, high-risk cohort, children had an increased risk of asthma at 6 years of age if they wheezed in the first 3 years of life with RSV [odds ratio (OR) 2.6], HRV (OR 9.8), or both HRV and RSV (OR 10).

***Respiratory syncytial virus***. By 1 year of age, 50–65% of children will have been infected with this virus, and by 2 years of age, nearly 100% has been infected (Openshaw, 1995). The exact mechanisms by which respiratory viral infection causes asthma exacerbation remains to be determined, but the respiratory viruses implicated in exacerbations have themselves been largely identified (**Figure 1**). The role of severe RSV infection as a risk factor for asthma in adulthood is less certain, but it is still under study. RSV is an important pathogen of young children and accounts for ~70% of severe infantile viral bronchiolitis and/or pneumonia cases, most of whom have wheezing, and it is the most common cause of hospital admission in the winter season during the first year of life (Blanken et al., 2013). Furthermore, this study showed the strongest evidence that human RSV-mediated bronchiolitis has long-term effects using palivizumab (a humanized monoclonal antibody against RSV F protein that prevents infection by RSV in infancy). In children under 5 years, RSV and PIV are the most common pathogens, whereas in older children, rhinovirus and influenza A virus are more prevalent (Beasley et al., 1988). Even in elderly persons, RSV causes pneumonia (Falsey et al., 2006), exacerbations of COPD, and acute deterioration in those with cardiac disease, and it contributes substantially to excess deaths in the winter season (Olszewska and Openshaw, 2009).

***Human rhinovirus***. Recent studies have identified infection with HRV as a predominant respiratory pathogen associated with asthma later in life (Kusel et al., 2007). HRV is the most important virus type associated with exacerbations of asthma leading to hospital admission in both adults and children (Johnston et al., 1996). Tan et al. (2003) reported that picornaviruses (rhinovirus/enterovirus) and AdV were most commonly identified in near-fatal asthma, whereas influenza virus predominated in COPD. Corne et al. (2002) found that the detection rates of HRV in asthmatic (10.1%) and healthy participants (8.5%) were similar, but the LRT symptoms were significantly more severe and longer-lasting in the asthmatic group than in the healthy group. Thus, HRV is the most common and important cause of exacerbation in both children and adults (Johnston et al., 1996; Rakes et al., 1999; Copenhaver et al., 2004; Message et al., 2008; Dougherty and Fahy, 2009; Olenec et al., 2010; Jackson et al., 2012). HRV can now be classified into three species (HRV-A, B, and C) based on their genetic properties (), while over 100 serotypes have been identified. Molecular epidemiological studies suggest that HRV-A and -C are the major prevalent species, with wide genetic divergence (Fujitsuka et al., 2011).

***Adenoviruses***. Adenoviruses are well known as a primary cause of acute respiratory infection, particularly in young children. AdVs are associated with up to 7% of virus-related asthma exacerbations (McErlean et al., 2010), and they cause a wide variety of clinical syndromes, such as diarrhea, keratoconjunctivitis, and hemorrhagic cystitis (Brodzinski and Ruddy, 2009). It has been demonstrated that 94% of children with refractory asthma has detectable AdV antigens, compared with 0% of controls (Macek et al., 1994; **Figure 1**).

***Parainfluenza virus***. As previously noted, PIV is one of the most common pathogens for asthma exacerbation in children under 5 years. In adults with asthma, PIV infections have also been commonly demonstrated in several longitudinal studies of RTIs, but they have been identified less commonly in studies of patients seen in the hospital or emergency department (Atmar et al., 1998).

***Other viruses***. Most asthma studies describe relatively low levels of influenza viruses in asthmatic patients with exacerbations, approximately 1–9% of all virus-related asthma exacerbations. Several studies indicated that human bocaviruses (Gendrel et al., 2007; Vallet et al., 2009) and HMPV (Williams et al., 2005; Ong et al., 2007) are associated with exacerbations of asthma, especially in children.

***Bacteria**. Mycoplasma pneumoniae *and* Chlamydophila pneumoniae *are found more frequently in the airways of patients with asthma than in healthy patients (Nisar et al., 2007), but their role in exacerbations is less clear (Xepapadaki et al., 2008). In previous studies, some have reported mycoplasmal infection in up to 25% of children with wheezing (Henderson et al., 1979) or identified it in 20% of exacerbations in asthmatic children requiring hospitalization and in 50% of children experiencing their first asthmatic attack (Biscardi et al., 2004). However, others have not been able to confirm these observations (Cunningham et al., 1998).

Other specific pathogens, including *Haemophilus influenzae*, *Streptococcus pneumoniae*, *Pseudomonas aeruginosa*, *Moraxella catarrhalis*, HRV, and RSV, have been shown to increase mucus secretion, which is recognized in asthma or COPD by characteristic goblet cell hyperplasia or enhanced mucus secretion (Fahy, 2002; Bisgaard et al., 2007; Kraft et al., 2008).

***Fungus***. Allergic bronchopulmonary aspergillosis (ABPA) is a unique form of asthma caused by colonization of the LRT (Vlahakis and Aksamit, 2001). ABPA is caused by an exaggerated T helper type 2 response to the ubiquitous mold *Aspergillus *spp., by which colonization leads to allergic and/or asthma symptoms (Edwards et al., 2012).

***Other factors***. The diverse etiologies for asthma exacerbation are well known, including viruses, allergens (dust mite, pollen, animal dander), smoking, gastroesophageal reflux disease, obesity, rhinosinusitis, stress, occupational exposures, hormones (menstrual asthma), drugs (acetylsalicylic acid, non-steroidal anti-inflammatory drugs, beta-blockers), exercise, and air pollutants. Physicians should be aware of these risk factors for asthma exacerbation (Dougherty and Fahy, 2009).

#### Mechanisms of viral-induced asthma exacerbations

Respiratory virus infection affects the pathogenesis of asthma. Bronchial epithelial cells are at the site of respiratory virus infection and replication. Respiratory virus infection induces production of various cytokines or chemokines and causes injury to epithelial cells or disruption of tight junctions. This inflammatory process may be amplified by intrinsic factors (susceptibility gene, family history of atopy, lung development) or environmental factors (respiratory virus infection, allergen exposure, smoking, and air pollutants, etc.; Hashimoto et al., 2008; Dougherty and Fahy, 2009). Some studies showing a deficiency in interferon (IFN)-β and IFN-λ production in response to HRV inoculation in airway epithelial cells cultured from asthmatic versus normal subjects (Holtzman, 2012) suggested that asthmatic patients have deficient IFN-β, IFN-λ, and perhaps some of the IFN-αs, but the precise mechanism or mechanisms behind deficient IFN production in these patients remain unknown.

#### Virus-associated clinical symptoms and exacerbations of asthma

In general, upper respiratory tract (URT) symptoms include rhinorrhea, sneezing, blocked nose, sore throat, hoarse voice, head or face ache, chill, and fever, while LRT symptoms include symptoms such as wheeze, cough, shortness of breath, and chest tightness (Corne et al., 2002). Tan et al. (2003) reported that virus-positive patients had a significantly increased frequency of URT symptoms of rhinorrhea, sore throat, fever, chills, and malaise. Nicholson et al. (1993) reported that, in adults with asthma, about a quarter of laboratory-confirmed viral and chlamydial acute upper respiratory infections was associated with mean decreases in peak expiratory flow of > 50 L/min, and half was associated with mean decreases of >25 L/min. The report also noted that respiratory pathogens were implicated in almost half of the most severe asthma exacerbations with a > 50 L/min mean decrease in peak expiratory flow. Viral infections have been shown to enhance both the reactivity of the lower airway and the magnitude of bronchoconstriction in response to inhaled contractile substances in asthma. The latter effect can persist for several weeks after infection, presenting as LRT symptoms (Cheung et al., 1995) accompanied by a decrease in peak expiratory flow. Thus, physicians should be aware of decreased peak expiratory flow, URT, or LRT symptoms associated with viral infections.

#### Treatment

The term “virus-induced exacerbation” is not uncommon, but only a small number of prospective studies have been conducted so far (Nicholson et al., 1993; Johnston et al., 1995). Importantly, respiratory infections do not always result in an exacerbation, and there is little evidence that treating or preventing the infection may cure or prevent an exacerbation (Xepapadaki and Papadopoulos, 2010). However, another study found that URT infections were strongly associated with exacerbations of asthma leading to hospital admission, in both adults and children (Johnston et al., 1996), and they may have contributed to asthma mortality, especially in the setting of hospital admission. Specific anti-viral therapies have not been established except for influenza viral infection, which have been recommended for persons with asthma or COPD. Furthermore, regarding preventive therapy for RSV, palivizumab as described above is now commercially available, and it might be appropriate for infants and young children with congenital heart disease, bronchopulmonary dysplasia, and prematurity before 35 weeks of gestation (Dawson-Caswell and Muncie, 2011). Blanken et al. (2013) stated that palivizumab treatment in healthy preterm infants born at a gestational age of 33–35 weeks reduced the number of wheezing days during the first year of life.

In this regard, several therapeutic strategies would need to be taken early in the course of infection to maximize the effects of treatments such as systemic corticosteroids, antibiotics if necessary, and short-acting β-agonist inhalers (SABAs), followed by inhaled corticosteroid (ICS) and long-acting β-agonist combination (LABA) therapy. Kerstjens et al. (2012) reported that additive long-acting muscarinic antagonist (LAMA) therapy with tiotropium (known as a cornerstone of COPD treatment) significantly increased the time to the first exacerbation and improved FEV_1.0_ in poorly controlled asthmatic patients with standard therapy (ICS and LABA). Similarly, tiotropium improved lung function and reduced the chance of rescue inhaler (SABA) in patients with overlap syndrome (Magnussen et al., 2008).

## VIRUS-INDUCED EXACERBATIONS IN CHRONIC OBSTRUCTIVE PULMONARY DISEASE

### DEFINITION OF EXACERBATION IN COPD

Exacerbation of COPD is an event characterized by an acute increase in respiratory symptoms beyond normal day-to-day variation (Vestbo et al., 2013). Clinicians and researchers should always keep in mind that exacerbations of COPD are neither defined consistently nor matched in individual studies. Definitions of exacerbations are roughly divided into two groups, event-based exacerbations and symptom-based exacerbations, depending on the patients’ symptoms or clinical events, respectively. Symptoms were defined and include dyspnea, cough, and sputum volume or purulence. Clinical events were defined as a status requiring additional treatments such as systemic antimicrobials or steroids with or without admission. Diseases such as pneumonia, congestive heart failure, and pulmonary embolism that mimic and/or aggravate exacerbations were generally excluded from exacerbations of COPD.

### CLINICAL IMPORTANCE OF EXACERBATION

The clinical course of COPD, as well as that of asthma, is punctuated by exacerbations, which are characterized by sudden symptom worsening beyond the expected daily variation. Exacerbations are important events in the clinical course of COPD, because they are associated with significant mortality. The in-hospital mortality rate of patients admitted to the hospital with exacerbations of COPD was 8%, increasing to 23% after 1 year of follow-up (Groenewegen et al., 2003). Exacerbations are correlated with accelerated loss of lung function and quality of life and increased healthcare costs (Seemungal et al., 1998; Donaldson et al., 2002; Miravitlles et al., 2002).

### FREQUENCY OF EXACERBATIONS

Previous studies showed that the annual rate of event-based exacerbations of COPD was 0.85–1.30 per patient per year (Calverley et al., 2007; Tashkin et al., 2008; Wedzicha et al., 2008; Seemungal et al., 2009; Hurst et al., 2010). The INSPIRE study showed that the rate of symptom-based exacerbations was about two times as high as that of event-based exacerbations (Seemungal et al., 2009). In the ECLIPSE study, the exacerbation rates were 0.85 per person for patients with moderate disease (GOLD stage 2) and 2.00 for those with very severe disease (GOLD stage 4; Hurst et al., 2010). Thus, the rate of exacerbation seems to depend on the disease severity (GOLD stage). However, it is particularly worth noting that the ECLIPSE study showed a subgroup of COPD patients that appeared to be susceptible to exacerbations, irrespective of GOLD stage. Other factors for exacerbations were several environmental factors, such as seasons or inhalation of harmful substances, and epidemic peaks in exacerbations of COPD were noted in both late fall and winter in the same fashion as in adult asthma (Johnston and Sears, 2006).

### CAUSES OF EXACERBATIONS

It has been reported that exacerbations are predominantly caused by bacterial and viral respiratory infections (**Figure 2**), and air pollution has a minor contribution. Previous studies showed that bacteria (*H. influenzae*, *S. pneumoniae*, *Moraxella catarrhalis, *and* P. aeruginosa*) and respiratory viruses (HRV, RSV, influenza virus, HMPV, coronavirus, and AdVs) were recognized during exacerbations. Bacteria, such as *H. influenzae*, *S. pneumoniae*, *Moraxella catarrhalis,* and *P. aeruginosa* were also detected in stable patients (Sapey and Stockley, 2006; Sethi and Murphy, 2008). When strains of bacteria are changed among the same species or there is emergence of other bacteria, this might cause inflammation in the lung in COPD patients and result in exacerbation (Sethi et al., 2002). The role of atypical respiratory pathogens, such as *Mycoplasma pneumoniae* and *C. pneumoniae*, in exacerbations of COPD is poorly recognized (Sapey and Stockley, 2006; Sethi and Murphy, 2008; Perotin et al., 2013). On the other hand, Blasi et al. (2002) showed that *C. pneumoniae* may be associated with exacerbation of COPD. Viruses such as HRV, RSV, and influenza virus have a higher prevalence in patients with exacerbations of COPD than in stable patients (Rohde et al., 2003; Wilkinson et al., 2006a).

### ROLES OF RESPIRATORY VIRAL INFECTION IN COPD EXACERBATIONS

**FIGURE 4:**
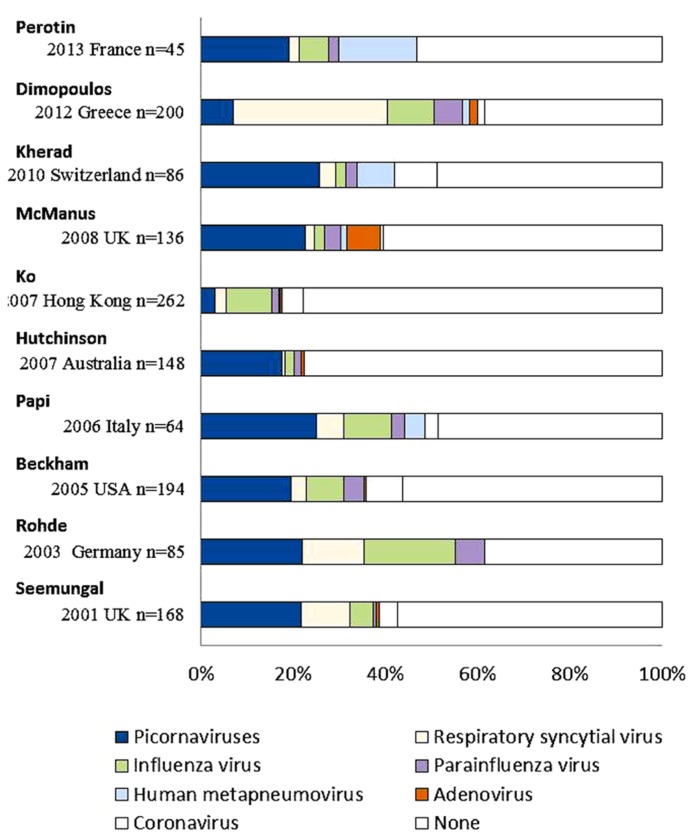
**Viral detection in exacerbations of COPD using PCR or RT-PCR methods in recent studies.** Picornaviruses include human rhinovirus and human enterovirus. Multiple viruses were detected in individual patients per one episode.

A few decades ago, it was considered that the role of respiratory viral infections was not a major cause in exacerbations of COPD because of the low sensitivity for viral detection, which depended on conventional technical methods such as viral culture or serological tests. However, recent studies have used new diagnostic technologies such as PCR or RT-PCR methods, which have a higher sensitivity for viral detection than conventional methods. Viral detections accounted for 22–57% of exacerbations of COPD in recent studies (**Figure 4**) using PCR or RT-PCR with observational periods of at least 1 year. The major viruses associated with exacerbations were HRV (3.1–26.6%), RSV (0.7–40.5%), and influenza virus (2.0–22.4%; Seemungal et al., 2001; Rohde et al., 2003; Tan et al., 2003; Beckham et al., 2005; Papi et al., 2006; Hutchinson et al., 2007; Ko et al., 2007; McManus et al., 2008; Kherad et al., 2010; Dimopoulos et al., 2012; Perotin et al., 2013).

Major respiratory viruses detected during exacerbations of COPD were HRV, RSV, and influenza virus, similar to those of asthma (Tan et al., 2003). Other viruses, such as PIV, human metapneumovirus, AdV, and coronavirus, were also noted in patients with COPD exacerbations. The proportion of viral-related exacerbations seemed to be similar among the various GOLD stages, while that of bacteria-related exacerbations increased with higher stage or a decrease in lung function (Dimopoulos et al., 2012).

#### HRV

Human rhinovirus was the most detected virus during exacerbations of COPD, but not only HRV infection alone but also co-infection with HRV and bacteria may cause exacerbations (Wilkinson et al., 2006b). Mallia et al. (2011) reported that experimental HRV infection showed more severe and prolonged lower respiratory symptoms, airway obstruction, and neutrophilic airway inflammation in COPD patients than in control subjects. They stated that rhinovirus infection led to elevation of neutrophil elastase, which is associated with reduction of antimicrobial peptides, such as secretory leukoprotease inhibitor and elafin. This reduction of antimicrobial peptides predisposes to secondary bacterial infection (Mallia et al., 2011, 2012).

Previous reports showed that COPD exacerbations may be associated with an impaired host response to HRV. For example, reduced IFN production was observed in COPD patients compared to control subjects, which may be associated with the mechanism of viral and subsequent bacterial infection related to severe exacerbations. Impaired humoral immunity was also related to exacerbation of COPD. Patients hospitalized with COPD exacerbations had lower serum levels of rhinovirus-specific antibodies than those not hospitalized with COPD exacerbations (Yerkovich et al., 2012). Thymic stromal lymphopoietin (TSLP) is a key pro-allergic cytokine that has recently been linked to asthma (Redhu and Gounni, 2012). In addition, genome-wide association studies showed that polymorphisms near or within the *TSLP *gene were associated with various allergic diseases, including bronchial asthma (Hirota et al., 2011; Ober and Yao, 2011). TSLP may contribute to exacerbations of the pathogenic effects of HRV infection via a Toll-like receptor (TLR)3-dependent pathway (Calven et al., 2012).

#### RSV

Respiratory syncytial virus has been detected in both stable and exacerbated cases of COPD. RSV detection in stable COPD patients might be associated with insidious airway inflammation and accelerated decline in FEV_1.0_ (Wilkinson et al., 2006a). However, this was not confirmed by another study (Falsey et al., 2006). RSV increases the expression of TLR3 on the surface of airway epithelial cells, which is associated with increased sensitization to double-stranded RNA and its related infections (Groskreutz et al., 2006).

#### Influenza virus

Influenza virus has been associated with mortality and morbidity in chronic lung diseases (Harper et al., 2009). A meta-analysis showed that influenza vaccination prevented exacerbations in COPD patients (Poole et al., 2000) and reduced the mortality and morbidity in elderly persons (Nichol et al., 2007). Anti-viral treatment such as neuraminidase inhibitors may reduce the severity of disease (Kaiser et al., 2003). Thus, treatment to prevent influenza has been recommended for COPD patients (Harper et al., 2009).

### ARE PATIENTS WITH COPD SUSCEPTIBLE TO RESPIRATORY VIRAL INFECTIONS?

Greenberg et al. (2000) investigated viral infections with or without COPD in a longitudinal cohort study. They demonstrated that annual symptomatic documented respiratory viral infection rates were similar for COPD and age-matched controls (0.45/year and 0.54/year, respectively). However, the COPD patients had more office visits and hospitalization than controls.

Of note, Mallia et al. (2011, 2012) confirmed these findings in a human experimental study; they found no significant differences in the frequencies of successful HRV infection between COPD and control groups when these groups were experimentally inoculated with HRV. Respiratory symptoms and a decline in lung function were more severe in the COPD group than in the control group.

### DIFFERENCES IN VIRAL AND NON-VIRAL EXACERBATIONS

Several studies have suggested that respiratory virus-associated exacerbations are more critical events than those due to other causes, in that viral-detected exacerbations showed a larger decline in lung function and longer recovery time than non-viral exacerbations (Seemungal et al., 2001; Bafadhel et al., 2011). As described in the chapter on HRV, respiratory viral infections themselves exacerbated COPD patients and tended to provoke secondary bacterial infections. Viral and sequential bacterial infections may be associated with severe respiratory symptoms (Wilkinson et al., 2006b; Harper et al., 2009; Mallia et al., 2012).

### MECHANISMS OF VIRUS-INDUCED COPD EXACERBATIONS

As shown in the **Table 1**, the pathological features of COPD are fibrosis around small airways involving several different cells (neutrophils, macrophage, CD8 lymphocytes) and destruction of lung parenchyma. Neutrophils have been found to be associated with both stable and exacerbated COPD (Hogg et al., 2004; Papi et al., 2006).

Changes in neutrophil counts during exacerbations in both sputum and peripheral blood have been found to be related to the FEV_1.0_ value. Levels of tumor necrosis factor-alpha and interleukin (IL)-8 in sputum were associated with neutrophilic inflammation (Keatings et al., 1996). Especially in patients who suffered from frequent exacerbations, they had persistently higher systemic IL-6 and C-reactive protein (CRP) levels, which may explain the greater decline in lung function (Perera et al., 2007).

Inflammatory cytokines in sputum during exacerbations have been found to be elevated regardless of whether the infection is viral or bacterial (Aaron et al., 2001), and their levels were higher with exacerbations than when stable. Eosinophils are considered characteristic cells in asthma, but they are also detected with exacerbations of COPD (Saetta et al., 1994). Indeed, Papi et al. (2006) demonstrated that virus-associated exacerbations in COPD patients were related to increased eosinophil counts and the level of eosinophil cationic protein. Furthermore, Bafadhel et al. (2011) showed that serum C-X-C motif chemokine 10 (CXCL10) is implicated as a more potent predictive maker for virus-associated exacerbations, and it is known as IFN-λ-induced protein 10.

### TREATMENT OF STABLE AND EXACERBATION STATES

Inhaled bronchodilators, LAMA and LABA, are the main pharmacological therapies in stable COPD patients (Tashkin et al., 2008; Wedzicha et al., 2008; Vestbo et al., 2013). Although Vogelmeier et al. (2011) reported that the tiotropium (LAMA)-treated group had a lower exacerbation rate than the salmeterol (LABA)-treated group in their head-to-head study, both LAMA and LABA treatments decreased exacerbation rates and improved lung function or health-related quality of life. Tashkin et al. (2009) found that combination LAMA/LABA therapy improved pulmonary function (FEV_1.0_) and respiratory symptoms better than LAMA therapy alone. ICS, the main treatment for asthma, is also prescribed in COPD patients and may reduce airway inflammation and decrease exacerbation rates only in moderate and severe COPD patients (Calverley et al., 2007). Treatment with macrolide antibiotics has been reported to prevent COPD exacerbations and improve patient quality of life and symptoms, especially in patients who have frequent exacerbations (Albert et al., 2011; Yamaya et al., 2012a), although this intervention could lead to unfavorable events such as increasing the prevalence of macrolide-resistant pathogens or cardiac toxicity.

It has been estimated that most exacerbations of COPD are due to respiratory viral and/or bacterial infections. Thus, the major pharmacological components of managing exacerbations of COPD include SABAs, short-course systemic glucocorticoids, and antibiotics (Vestbo et al., 2013). However, anti-viral therapies are rarely prescribed, because specific anti-viral therapies do not exist, except for influenza virus and RSV. Treatment for influenza appears appropriate in patients with COPD (Harper et al., 2009), while the utility of treatment for RSV has not been confirmed in adults. It is doubtful that systemic corticosteroid treatment affects the clinical course of respiratory viral infections. Lee et al. (2011) showed that short-course systemic steroid treatment did not affect viral load or shedding, and humoral immunity may be diminished by steroid treatment.

Some research has shown that LAMA may affect viral infections. Tiotropium, one of the LAMAs, may inhibit HRV and RSV infections by reducing the levels of intercellular adhesion molecule-1, which is the binding site for most HRVs (Iesato et al., 2008; Yamaya et al., 2012b).

## SUMMARY AT A GLANCE

The associations between virus infections and asthma and/or COPD were reviewed, and the significance of viral infections, as well as their effect on the clinical course, was discussed.

(1) HRV and RSV are major causes of exacerbations both in asthma and COPD patients.

(2) The frequency of viral detection in both asthma and COPD patients appears to be similar to that of healthy subjects, but the effect on their clinical course is different; asthma and COPD patients tend to have more severe or persistent respiratory symptoms or decreases in pulmonary function, and mortality may be increased.

(3) Since discrimination between asthma and COPD is difficult, especially during exacerbations, whenever clinicians encounter patients in whom obstructive airway disease is suspected, multidisciplinary assessment is required for diagnosis.

(4) The clinical findings of both asthma and COPD, so-called “overlap syndrome,” are commonly recognized in general practice, and virus-associated exacerbations in this disease may lead to a poor prognosis.

## Conflict of Interest Statement

The authors declare that the research was conducted in the absence of any commercial or financial relationships that could be construed as a potential conflict of interest.

## Footnotes

1

2

## Abbreviations

ABPA: allergic bronchopulmonary aspergillosis
AdVs: adenoviruses
BAL: bronchoalveolar lavage
COPD: chronic obstructive lung disease
CRP: C-reactive protein
FEV_1.0_: forced expiratory volume in 1 second
GOLD: Global initiative for chronic obstructive lung disease
HMPV: human metapneumovirus
HRV: human rhinovirus
ICS: inhaled corticosteroid
IFN: interferon
IL: interleukin
LABA: long-acting β-agonist combination
LAMA: long-acting muscarinic antagonist
LRT: lower respiratory tract
NPS: nasopharyngeal swabs
NPW: nasopharyngeal washings
OPS: oropharyngeal swabs
PEFR: peak expiratory flow rate
PIV: parainfluenza virus
RSV: respiratory syncytial virus
RTI: respiratory tract infection
RT-PCR: reverse-transcriptase polymerase chain reaction
SABA: short-acting β-agonist inhaler
TLR: toll-like receptor
TLSP: thymic stromal lymphopoietin
URT: upper respiratory tract
